# Self-Supervised Wavelet-Based Attention Network for Semantic Segmentation of MRI Brain Tumor

**DOI:** 10.3390/s23052719

**Published:** 2023-03-02

**Authors:** Govindarajan Anusooya, Selvaraj Bharathiraja, Miroslav Mahdal, Kamsundher Sathyarajasekaran, Muniyandy Elangovan

**Affiliations:** 1 Vellore Institute of Technology, Chennai Campus, Chennai 600127, India; 2 Department of Control Systems and Instrumentation, Faculty of Mechanical Engineering, VSB-Technical University of Ostrava, 17. Listopadu 2172/15, 708 00 Ostrava, Czech Republic; 3 Department of R&D, Bond Marine Consultancy, London EC1V 2NX, UK

**Keywords:** semantic image segmentation, self-supervised wavelet-based attention network (SSW-AN), attention mechanisms, self-supervised attention block (SSAB), Wavelet transform

## Abstract

To determine the appropriate treatment plan for patients, radiologists must reliably detect brain tumors. Despite the fact that manual segmentation involves a great deal of knowledge and ability, it may sometimes be inaccurate. By evaluating the size, location, structure, and grade of the tumor, automatic tumor segmentation in MRI images aids in a more thorough analysis of pathological conditions. Due to the intensity differences in MRI images, gliomas may spread out, have low contrast, and are therefore difficult to detect. As a result, segmenting brain tumors is a challenging process. In the past, several methods for segmenting brain tumors in MRI scans were created. However, because of their susceptibility to noise and distortions, the usefulness of these approaches is limited. Self-Supervised Wavele- based Attention Network (SSW-AN), a new attention module with adjustable self-supervised activation functions and dynamic weights, is what we suggest as a way to collect global context information. In particular, this network’s input and labels are made up of four parameters produced by the two-dimensional (2D) Wavelet transform, which makes the training process simpler by neatly segmenting the data into low-frequency and high-frequency channels. To be more precise, we make use of the channel attention and spatial attention modules of the self-supervised attention block (SSAB). As a result, this method may more easily zero in on crucial underlying channels and spatial patterns. The suggested SSW-AN has been shown to outperform the current state-of-the-art algorithms in medical image segmentation tasks, with more accuracy, more promising dependability, and less unnecessary redundancy.

## 1. Introduction

In the field of medical image processing, segmentation has lately gained popularity. Segmentation is utilized to facilitate diagnosis and treatment planning. Because it labels every pixel in an image with a category and is therefore more precise and efficient than other approaches, CNN-based methods for semantic segmentation have recently gained in popularity. Semantic Broadcast News Networks (CNNs) aim to solve problems with medical segmentation tasks [1]. Semantic object segmentation, a crucial component of medical image analysis, has already been widely used to automatically identify regions of interest in 3D medical images, such as cells, tissues, or organs [2,3]. Convolutional networks’ recent progress has resulted in major developments in medical semantic segmentation, that provide cutting-edge outcomes in a number of real-world applications. However, medical segmentation issues are notoriously expensive to resolve, and labelled data are frequently needed for convolutional neural network training [4]. Segmentation is a critical step in the image-processing process with numerous potential applications in fields as varied as scene understanding, medical imaging analysis, image-guided treatments, radiation protection, and enhanced radiological diagnostics. A picture is segmented when it is split up into a number of distinct, non-overlapping areas, the union of which produces the original image [5]. Medical imaging technologies have developed quickly and been widely used, which has led to the collection of a lot of data that can be used for analysis [6]. To enable doctors to accurately understand the diagnosis information included in the images and treat a large number of patients more effectively, the need for automated processes that can quickly and impartially evaluate these medical images is growing [7,8].

**Figure 1:**
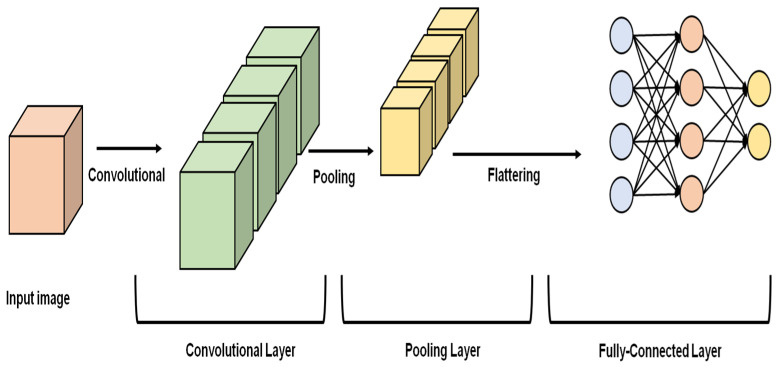
Convolution neural network.

Alalwan et al. [9] recommended the 3D-DenseUNet-569 system for the segmentation of tumors and the organ. Experimental results on the manufacturing LiTS dataset validate the efficiency and usefulness of the 3D-DenseNet-569 model. 3D-DenseUNet-569 has a deeper network and fewer trainable parameters. Instead of using traditional convolution, DS-Conv is employed. DS-Conv decreases GPU memory and computational expenses. According to Fang et al. [10], a difference Minimization Network (DMNet) is the new end-to-end method provided for semi-supervised semantic segmentation. The approach outperforms supervised and semi-supervised baselines in tests on two cancer datasets. To help reduce data inequalities, Rezaei et al. [11] suggested a novel architecture for adversarial generation. The generative network’s incorrect positive and negative mask predictions teach the refinement network how to make accurate predictions. The output from the three networks are the semantic segmentation masks that are produced. Rezaei et al. [12] proposed RNN-GAN as a method of overcoming the information imbalance inherent in medical picture feature extraction, where the amount of object pixels of interest is often significantly less than that of the surroundings. Models that are biased in favour of healthy data due to unbalanced data are not suitable for clinical applications. Jiang et al. [13] used CNN to segment medical images semantically. Recent deep learning developments allow comprehensive image analysis using fully convolutional neural networks. The convolutional neural network SMILE was described by Petit et al. [14] and can learn from imperfect input. SMILE recognizes ambiguous labels during training and ignores them so that misleading or noise information is not spread. Experimental tests of the proposed semantic segmentation method on pictures of the liver, stomach, and pancreas show that, despite having 70% of its annotations missing, SMILE achieves results comparable to those of a baseline trained with all of the ground truth annotations. Karayegen et al. [15] proposed an approach for automatically segmenting brain tumors from sets of 3D Brain Tumor Segmentation (BraTS) image data created using four different imaging modalities. As a consequence, it is possible to accurately diagnose brain tumors using semantic segmentation measures and 3D imaging. To solve the problems of failure and anomaly detection for semantic segmentation, Xia et al. [16] proposed a two-module unified solution. On Cityscapes, pancreatic tumor segmentation in MSD, and Street Hazard’s anomaly segmentation. Wang et al. [17] suggested using a technique that integrates Vision Transformer with a semi-supervised strategy for improved medical image semantic segmentation. There are two primary models in this framework: one for the instructor and one for the learner. The teacher model imparts its knowledge to the student model, which then uses the absorbed picture features to further its learning. In histopathological whole-slide semantic segmentation, to integrate context and specificity, Van Rijthoven et al. [18] created HookNet, a model that employs numerous branches of encoder-decoder convolutional neural networks. They made HookNet available to the general public by releasing its source code1 and hosting its web-based applications on the grand-challenge.org platform at no cost. The issue of semantic segmentation in prenatal ultrasound volumes was investigated by Yang et al. [19]. The method has been thoroughly tested on internal big data sets and shows outstanding segmentation performance, fair agreement with expert measurements, and strong reproducibility against scanning variances, making it hopeful for the future of prenatal ultrasound examinations. Ali et al. [20] proposed combining a 3D CNN with a U-Net segmentation network into an ensemble as a substantial but simple combinative strategy that yields more precise predictions. On the BraTS-19 testing data, both models were trained individually and assessed to provide segmentation maps that significantly varied from one another in terms of segmented tumor sub-regions. Kumar et al. [21] implemented a reliable crude k-means algorithm. Sensitivity, specificity, and accuracy are used to assess how well the given approach performs. The experimental findings demonstrate that our suggested approach produced superior outcomes versus earlier research. Wang et al. [22] presented the TransBTS network, a specialized core network on the encoder-decoder architecture, and used Transformers in 3D CNN for the first time for MRI brain tumor segmentation. The volume spatial feature maps are extracted by the encoder using 3D CNN before the local 3D background data are captured. CNNs are composed of convolution layers with convolutional weights and biases similar to those found in neurons. The fundamental components of CNNs are the convolutional layer and the fully connected layer Figure 1.

Wadhwa et al. [23] discussed a comprehensive analysis of the literature on current techniques for segmenting brain tumors from brain MRI data. Modern techniques are used, and their effectiveness and quantitative analysis are included. With the most current contributions from several academics, different picture segmentation techniques are briefly discussed. Zhao et al. [24] examined the various methods used for 3D brain tumor segmentation using DNN. These approaches for data processing, such as collecting data, randomized image training, and semi-supervised learning, are divided into three primary categories; model-building techniques such as architectural design and result fusing, as well as process-optimization techniques such as heating learning and multi-task learning. Liu et al. [25] used a heuristic approach to find a mathematical and geometric solution to this issue in order to enhance the segment of overlapping chromosomes. The issues that arise and its solutions are provided as graphically depicted interpretable image features starting with chromosomal images, which assists in a better comprehension of the process. Bruno et al. [26] proposed a Deep Learning (DL)-based method for the semantic segmentation of medical images. Specifically, they employed ASP to encode past medical knowledge, developing a rule-based model for disabling all permissible classes and correct place concatenations in medical picture data. The results of an experimental study are presented to assess the practicability of the approach. Emara et al. [27] suggested LiteSeg, a compact framework for semantic image segmentation. By using depth-wise separable convolution, short and long residual connections, and the Atrous Spatial Pyramids Pooling module (ASPP), they evaluate a faster and more efficient model. To improve the multi-scale processing capability of neural networks, Qin et al. [28] suggested the autofocus convolutional layer for semantic segmentation. Fang et al. [29] developed a system for multi-modal brain tumor segmentation that combines hybrid features from several modalities while using a self-supervised learning approach. The technique uses a fully convolution neural network as its foundation. Ding et al. [30] proposed a brand-new multi-path adaptive fusion network. To reserve and propagate more low-level visual elements more efficiently, they explicitly apply the concept of skip connection in ResNets to the dense block. The network has achieved a contiguous memory mechanism by implementing directed links from the state of the previous dense block to all levels of the current dense block. Jiang et al. [31] proposed the MRF-IUNet multiresolution fusion MRI brain tumor segmentation technique, which is based on an enhanced inception U-Net (multiresolution fusion inception U-Net). The breadth and depth of the network are extended by adding inception modules to U-Net in place of the initial convolution modules. Zhou et al. [32] developed an attention-based multi-modality fusion network for segmenting brain tumors. The network incorporates a feature fusion block to combine the four features, four channel-independent encoding routes to separately extract features from four modalities, and a decoding path to eventually segment the tumor. Liu et al. [33] used low-level edge information as a precursor job to help with adaptation as it has a smaller cross-domain gap than semantic segmentation. So that the semantic adaption may be guided by spatial information, the exact contour is then given.

This article provides an interference-capable framework for unified picture fusion. The method is recommended in light of a brand-new issue in self-supervised picture reconstruction. In particular, it uses discrete wavelet transform to specifically decompose the image in the spectral domain, and then rebuild it using an encoder–decoder paradigm. We tested our algorithms on difficult tasks including MRI brain tumor segmentation, and found it to be very promising.

The article makes the following contributions—

- 1251 patients’ 3D MRI datasets were gathered from the BraTS dataset in the research.
- The Self Supervised Wavelet-based Attention Network (SSW-AN), which splits low-frequency and high-frequency data into four channels, employs the 2D Wavelet transform.
- We use self-supervised attention channels and spatial attention modules (SSAB).
- We give in-depth analysis of the scientific advancements achieved in the field of semantic image segmentation for natural and medical images.
- We discuss the literature on the various medical imaging modalities, including both 2D and volumetric images.

This research follows the following structure: The problem under examination is described in Section 2. Our strategy is described in Section 3. Section 4 provides a thorough discussion of the results. The final Section 5 offers the conclusion.

## 2. Problem Statement

Due to the increasing number of variations and the possibility of morphological changes between them, it can be difficult to identify symptomatic signals for therapeutic usage. Due to the lack of spatial information for an object’s texture, 2D photographs cannot help clinical diagnosis. 3D photos with spatial information are more qualified than 2D images for medical segmentation. 3D image segmentation algorithms are limited [34]. 3D medical images are volumetric, making segmentation efficacy and efficiency difficult to balance.

## 3. Methodology

### 3.1. Self-Supervised Wavelet-Based Attention Network

**Figure 2:**
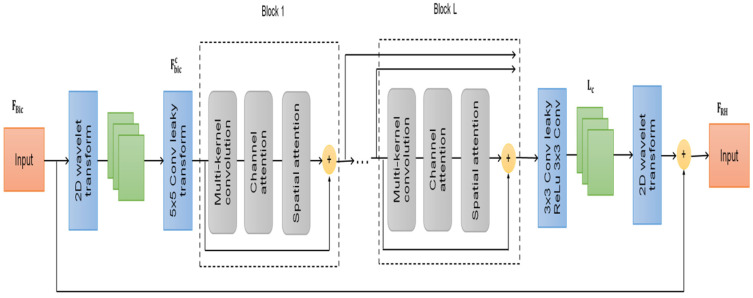
Self-Supervised wavelet-based attention network.

Self-supervised wavelet-based attention network is a well-known classical image-processing method for image analysis. First, low-pass and high-pass filters are applied to the image before it is half-down-sampled along columns. Then, two pathways are sent through “low-pass and high-pass filters in that order. Each of these bands represents a different type of data extracted from the original image, such as the mean, the verticals, the horizontals, and the diagonals. Each wavelet domain is half the size of the main band. The wavelet transform and its inverse are inevitable, ensuring the integrity of the data. Since the Wavelet transform can be used in reverse, our approach can easily recover the original residual image. Applying the Wavelet transform on the residual self-supervised image allows our model to predict four half-sized channels, or roughly four coefficients. The fact that our model’s underlying patterns are stored across four channels rather than in a single huge image greatly accelerates its learning time, as seen in Figure 2.

Specifically, the model $F_{\mathrm{bic}}^{c}\;\epsilon \;{\mathbb{R}}^{\frac{r}{2}\times \frac{c}{2}\times w}$ takes as input the two-dimensional wavelet transform using four coefficients to the bi-cubic image $F_{bic}^{c}{\mathbb{R}}^{r\times c}$. They are divided into four channels and shrunk in both the horizontal and vertical dimensions in preparation for training. In the first step, we utilize a fully connected layer to glean superficial information from the input:

(1) $$I_{0}=I_{\mathrm{ext}}\left(F_{\mathrm{bic}}^{c}\right)=\sigma \left(W\left(F_{\mathrm{bic}}^{c},5\times 5,w\right),\alpha \right)\epsilon \;{\mathbb{R}}^{\frac{r}{2}\times \frac{c}{2}\times 4}$$

where W($F_{\mathrm{bic}}^{c}$) stands for a full connection layer, r is the reduction ratio, c is the feature vectors, bic is bicubic interpolation, σ denotes a leaky rectified linear unit (ReLU) layer, w is the convolution layer, with a kernel size of 5 × 5 as well as a channel size of $\sigma \left(W\left(F_{\mathrm{bic}}^{c},5\times 5,w\right),\alpha \right)$, which means a linear unit layer with a leaky rectifier. For non-linear activation, we employ a leaky version of ReLU since $F_{\mathrm{bic}}^{c}$ by the Wavelet transform naturally includes negative 240 pixels. Be aware that bias is left out for concise notations. Due to the large quantity of data that is linked with digital photographs, the bicubic interpolation method is employed for higher interpolation quality.

Each of the model’s L consecutive, identical blocks features a cross fully connected layer, as well as a channel attention module, along with a spatial attention module, which together form every model’s attention architecture. For faster data transfer, we set up local skip connections between each block. So, we have arrived at the following:

(2) $$I_{f+1}=R_{f+1}\left(I_{f}\right)=i_{\mathrm{spa}}\left(i_{\mathrm{chn}}\left(I_{f}\right))\right)+I_{f},f=0,1,\ldots ,T-1$$

where T is successive identical blocks, $i_{\mathrm{chn}}$ denotes the multichannel channel attention function and $i_{\mathrm{spa}}$ denotes the spatial attention function. Keep in mind that each block’s output has the same dimension as its input.

We connect the data from every one of these blocks along the channel dimension to address the common gradient vanishing problem in neural network-based designs.

(3) $$I_{cat}=\left[I_{1},I_{2},\ldots ,I_{T}\right]\epsilon {\mathbb{R}}^{\frac{r}{2}\times \frac{c}{2}\times \mathrm{wT}}$$

It is possible to successfully back-propagate gradient information to the front of the network by using feature mappings from shallow layers to deep layers in forward computation. On the basis of empirical data, this paradigm may improve training convergence.

(4) $$I_{c}=W(\sigma (W\left(I_{cat},3\times 3,w\right),\alpha ),3\times 3,4)\epsilon {\mathbb{R}}^{\frac{r}{2}\times \frac{c}{2}\times 4}$$

The network is trained to produce an output $I_{c}$ that faithfully simulates the four Wavelet transform coefficients on the true residual image $F_{RH}-F_{bic}$. In the alternative, we can consider

(5) $$F_{RH}≃fmCL\left(I_{c}\right)+F_{bic,}$$

where fm is inverse discrete, the SSW-AN of the function is denoted by $fmCL\left(I_{c}\right)$. A sizable $F_{bic,}$ the model closes with residual connections to ensure that the network is being trained to identify residual items and not the RH image. This is how we implement global residual learning. This aids in strength training and quick convergence as standard tactics.

### 3.2. Channel Attention Module 

**Figure 3:**
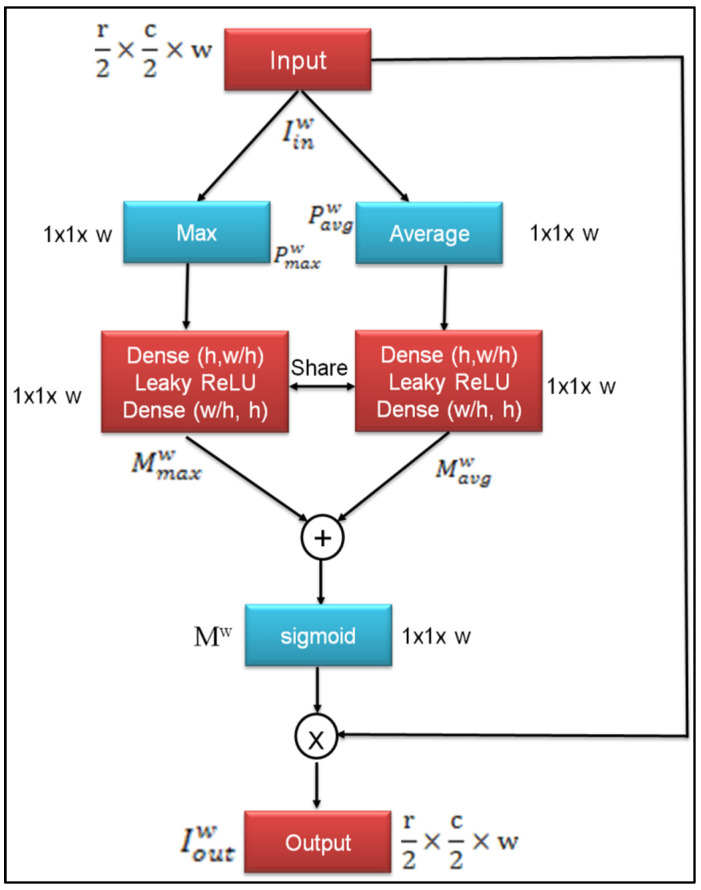
Channel attention module.

A higher dimensional space channels network may be thought of as a class-specific response and different semantic responses are coupled to one another. One may improve the visual features of certain semantics by highlighting the physical architecture of connectivity via the dependence between channel graphs. We make channel attention modules that will directly simulate the dependence between channels by determining the magnitude of any two channel correlations. Figure 3 depicts the structural arrangement of the channel attention module. The channel interdependencies that feature maps have been utilized in this module. The many channels that are essential to the computing process will be the main topic of this section.

In order to obtain spatial context information, the input feature $I_{out}^{w}$ is compressed using a maximum pooling operation and compression using an average pooling operation to generate spatial contextual data. This results in the generation of two vectors:

(6) $$\{\begin{array}{c} P_{max}^{w}=V\left(I_{in}^{w}{,}^{\prime }max^{\prime },axis=\left[0,1\right]\right)\epsilon {\mathbb{R}}^{\frac{r}{2}\times \frac{c}{2}\times w}\\ P_{avg}^{w}=V\left(I_{in}^{w}{,}^{\prime }avg^{\prime },axis=\left[0,1\right]\right)\epsilon {\mathbb{R}}^{\frac{r}{2}\times \frac{c}{2}\times w} \end{array}$$

where axis=[0,1] specifies that pooling occurs along the first two dimensions of the feature $I_{in}^{w}$, and max and avg stand for maximum and average pooling, respectively. After that, we feed two input vectors into two fully connected layers that are also connected via a shared parameter, and out of that we obtain two feature vectors. The elements of a vector can be thought of as labels for the various signals they represent.

(7) $$\{\begin{array}{c} M_{max}^{w}=C_{2}(\sigma (C_{1}\left(P_{max}^{w},w/h\right),\alpha ),w)\epsilon {\mathbb{R}}^{\frac{r}{2}\times \frac{c}{2}\times w}\\ M_{avg}^{w}=C_{2}(\sigma (C_{1}\left(P_{avg}^{w},w/h\right),\alpha ),w)\epsilon {\mathbb{R}}^{\frac{r}{2}\times \frac{c}{2}\times w} \end{array}$$

In this instance, $C_{1}$(.) and $C_{2}$(.) are shared by the two feature vectors. To reduce parameter overhead, the hidden layer size is set to w/h, where h is the reduction ratio. This plan allows for the use of relationships between channels by a simple calculation. $P_{max}^{w}$ and $P_{avg}^{w}$ description vectors are combined using an element-wise sum, then a sigmoid activation layer is applied:

(8) $$M^{w}=sig\left(M_{max}^{w}+M_{avg}^{w}\right)\epsilon {\mathbb{R}}^{\frac{r}{2}\times \frac{c}{2}\times w}$$

Finally, the element-wise product is used to apply the description vector $M^{w}$ to the input of this module, where each descriptor multiplies one feature map, denoted as

(9) $$I_{out}^{w}=M^{w}{}^{\circ}I_{in}^{w}\epsilon {\mathbb{R}}^{\frac{r}{2}\times \frac{c}{2}\times w}$$

where $M^{w}{}^{\circ}I_{in}^{w}$ stands for the product of the elements individually. Take into account that the dimensions of both the input and the output are the same. Therefore, it is straightforward to add this module to the standard Classier.

### 3.3. Spatial Attention Module 

**Figure 4:**
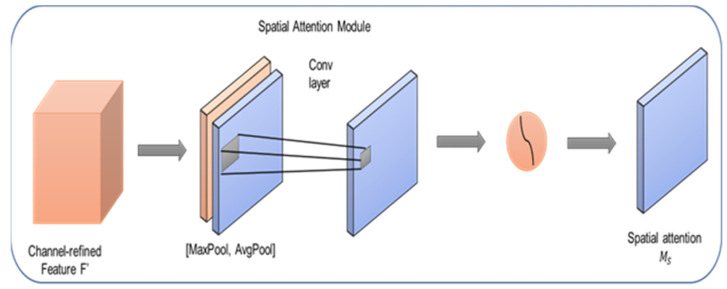
Spatial attention module.

The spatial attention module applies depending on spatial focus to each feature plane in an effort to increase feature learning for suitable locations. The spatial attention module develops responsive image features by amplifying significant locations inside each feature plane, improving the depth of characteristics for mild diseases and the value discrepancy between diseased and pre regions. Figure 4 shows how the spatial attention module uses spatial relationships between features to guide focus. When compared to channel attention, spatial attention narrows in on the different layers that reveal the most useful information.

Max pooling and average pooling methods squeeze the input feature $I_{in}^{4}$ in along the channel axis, producing two 2D attention maps:

(10) $$\{\begin{array}{c} D_{max}^{o}=V\left(I_{in}^{o}{,}^{\prime }max^{\prime },axis=2\right)\epsilon {\mathbb{R}}^{\frac{r}{2}\times \frac{c}{2}\times 1}\\ D_{avg}^{o}=V\left(I_{in}^{o}{,}^{\prime }{avg}^{\prime },axis=2\right)\epsilon {\mathbb{R}}^{\frac{r}{2}\times \frac{c}{2}\times 1} \end{array}$$

After that, a convolutional layer with a 7 × 7 kernel size is applied to combine and fuse them. The attention map is normalized to [0, 1] and nonlinearity is introduced using the sigmoid function:

(11) $$\{\begin{array}{c} D_{max}^{o}=V\left(I_{in}^{o}{,}^{\prime }max^{\prime },axis=2\right)\epsilon {\mathbb{R}}^{\frac{r}{2}\times \frac{c}{2}\times 1}\\ D_{avg}^{o}=V\left(I_{in}^{o}{,}^{\prime }{avg}^{\prime },axis=2\right)\epsilon {\mathbb{R}}^{\frac{r}{2}\times \frac{c}{2}\times 1} \end{array}$$

This module’s input is multiplied by the interest image element by element; this process is analogous to channel attention, in which each image value is used to multiply the elements at the appropriate locations of all wavelet coefficients.

(12) $$I_{out}^{o}=D^{o}I_{in}^{o}\epsilon {\mathbb{R}}^{\frac{r}{2}\times \frac{c}{2}\times w}$$

Ensure that both the input and the output have the same dimensions. Thus, this extension can be used in tandem with the standard classifier.

## 4. Loss Function

The image weight vector loss is the most often used loss function for the process of segmenting images [35]. A loss function tells the model how close it is to the ideal version parameters during supervised training. Weight vector loss is the most used loss function. Medical photographs often only show a tiny portion of the objects, such as the optic disc and retinal veins. For such applications, the weight vector loss is not the best option. In the part that follows, comparative tests and discussions are also carried out. When ground truth is known, segmentation performance is often evaluated using the Dice coefficient as a measure of overlap, as in Equation (13):

(13) $$K_{dice}=1-\overset{L}{\underset{l}{\sum }}\frac{2\omega_{l}\sum_{g}^{P}n\left(l,g\right)i\left(l,g\right)}{\sum_{g}^{P}n_{\left(l,g\right)}^{2}+\sum_{g}^{P}i_{\left(l,g\right)}^{2}}$$

N stands for the pixel number, while the variables $n_{\left(l,g\right)}\epsilon \left[0,1\right]$ and $i_{\left(l,g\right)}\epsilon \left[0,1\right]$ are the estimated likelihood and class k’s ground truth label, respectively. The formula $\sum_{l}\omega_{l}=1$ is the class weight, and K is the class number. In our paper, $\omega_{l}=\frac{1}{l}$ was determined experimentally. This is the definition of the final loss function. The final loss function is defined as Equation (14):

(14) $$L_{loss}=L_{dice}+L_{reg}$$

*L_reg_* stands for the regularization loss used to prevent overfitting. Medical picture segmentation problems include cell contour segmentation, lung segmentation, retinal vascular identification, and optic disc segmentation.

(15) $$loss=1-\frac{2\times \sum_{g=0}^{P}\left(\left(n_{g}i_{g}\right)\right)}{\sum_{g=0}^{P}n_{y}^{2}+\sum_{g=0}^{P}I_{y}^{2}}$$

Weighting was applied to the $i_{g}$ portion since it correlates to the brain tumor lesion region, and the ratio of predicted outcomes to real values in the loss function was 1:3. For the true value distribution, the loss function’s loss coefficient is higher. It can enhance the network’s characteristic learning of the brain tumor lesion area, weaken the distribution of the network’s loss value to the non-tumor area, and lessen the interference of the brain MRI background image on the characteristic learning of the lesion area, all of which will increase the network’s detection accuracy.

## 5. Results and Discussion

In MATLAB/Simulink, the proposed model is activated, and its efficacy is compared to that of existing models like Deep Convolutional Neural Networks (DCNN), Attention-Based Semi-Supervised Deep Networks (ASDNet), Deep Neural Networks (DNN), Global Context Network (GCN) and Nested Dilation Network (NDN) are compared with the proposed method. Accuracy, precision, recall, specificity, sensitivity, and MSE were analyzed using suggested and existing methods. 

### 5.1. Dataset

The BraTS challenge includes an image-annotated 3D MRI dataset from medical professionals, enabling the assessment of cutting-edge methods for semantics segmentation of brain tumors [36].

**Figure 5:**
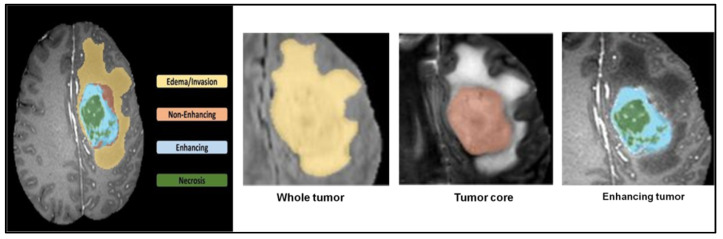
3D MRI brain tumor semantic segmentation.

Figure 5 depicts the typical results of segmentation for all semantic classes. The T2-FLAIR picture shows the tissue around the cystic/necrotic parts of the core as yellow, while the active tumor features are seen as light blue on the T1Gd image (green). Combining the ED, NET, NCR cores, and AT segmentations results in labeling for the distinct tumor sub-regions in the colors yellow, red, green, and blue (blue). For the BraTS 2021 test training dataset, 1251 people were included, each with their own 3D MRI of one of four possible types. All native (T1), post-contrast (T1Gd), T2-weighted (T2), and T2-FLAIR images are down-sampled to a resolution of 1 mm on a 1 mm grid and undergo skull-stripping, stiff orientation, and down-sampling. The input image’s dimensions are 240 pixels wide and 155 pixels high. Different types of MRI scanners were used to procure the data. The enhancing tumor, the edematous tissue surrounding it, and the necrotic, non-enhancing core of the tumor all have clear boundaries. Whole tumor (WT), tumor core (TC), and enhancing tumor (ET) are all terms that were derived from the annotations.

**Figure 6:**
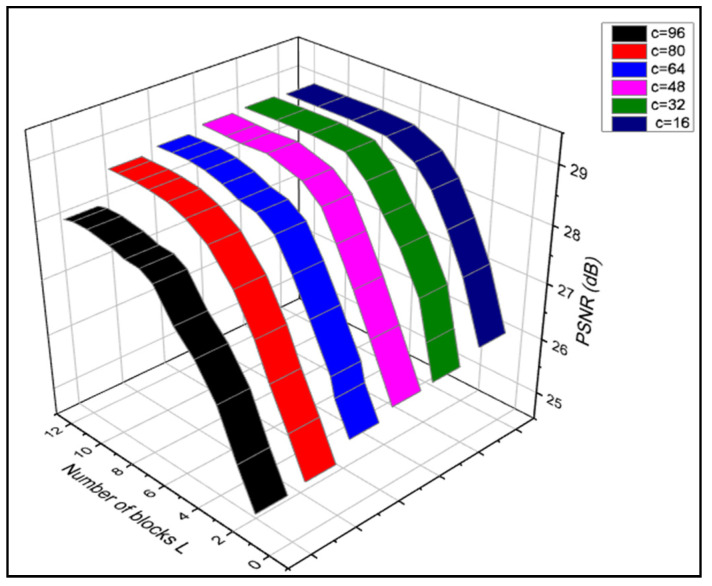
Peak signal noise ratio.

Peak signal-to-noise ratio (PSNR) is a popular statistic for assessing the efficacy of the reconstructed picture. This is consistent with our belief that bigger, deeper networks perform better because they are better equipped to learn. The outcomes of our technique for adjusting the number of blocks L and the channel width C are shown in Figure 6. We observe that the model performs better when given 385 blocks of L using a rising metric. This confirms our intuition that more complex networks are better able to learn and adapt. PSNR stabilizes because the performance is not necessarily improved over deep structures, which are also highly challenging to train. In addition, PSNR gradually rises as c grows bigger. However, increasing the value of c will result in a noticeable increase in the number of parameters and computing load. To find a good compromise between performance and model size, we settle on c=64, as shown in Table 1.

The degree to which a statement is true can be determined by dividing the total number of statements by the number of accurate classifications. The accuracy of this method is dependent on the classifier’s ability to correctly identify normal and pathological brain states. In statistics, precision means

(16) $$Accuracy=\frac{\mathrm{TP}\;+\;\mathrm{TN}}{\mathrm{TP}\;+\;\mathrm{TN}\;+\;\mathrm{FP}\;+\;\mathrm{FN}}$$

where TP represents the proportion of cases when abnormalities were properly diagnosed; FP is the total count of abnormal images that were incorrectly classified; TN represents the percentage of samples that were accurately labeled as normal; FN is that often-abnormal images were first classified as normal.

**Figure 7:**
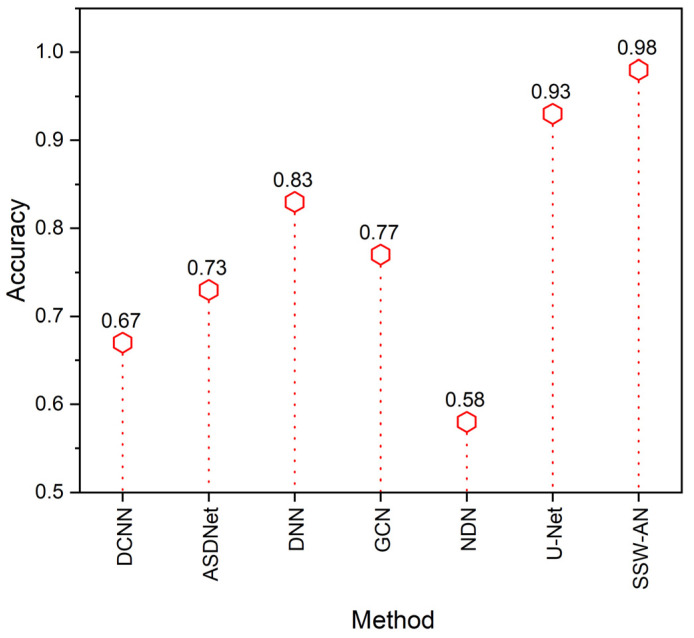
Accuracy of various methods. Current SSW-AN is compared with DCNN [37], ASDNet [38], DNN [35], GCN [39], NDN [40] and U-Net [41].

Figure 7 depicts the comparison of accuracy. The proposed SSW-AN was revealed to be more accurate than the existing techniques, including DCNN, ASDNet, DNN, GCN, and NDN.

**Figure 8:**
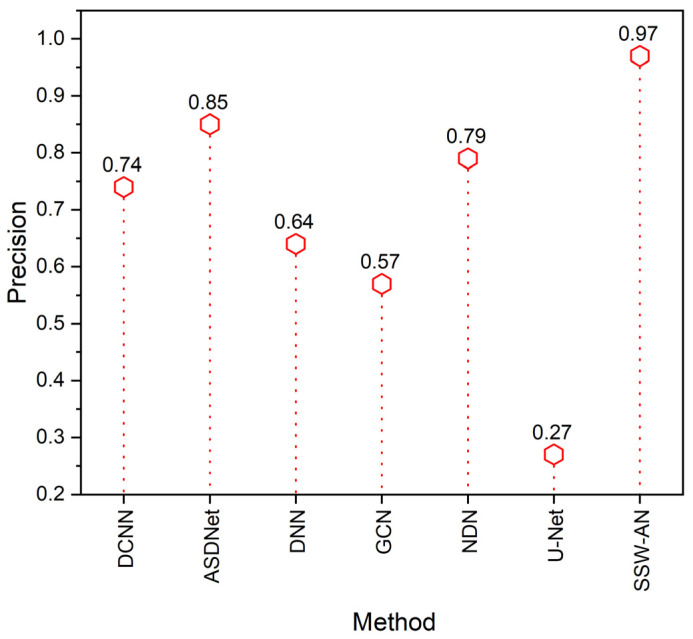
Precision of various methods. Current SSW-AN is compared with DCNN [37], ASDNet [38], DNN [35], GCN [39], NDN [40] and U-Net [41].

Comparison of accuracy is shown in Figure 8. Utilizing the statistic known as positive predictive value (PPV), precision may also be measured. The number of precise class predictions from a given sample is a measure of precision. In other words, it compares actual results to results that were expected. The formula described below can be used to figure out how precise an observation is:

(17) $$\mathrm{Precision}=\frac{\mathrm{TP}}{\;\mathrm{TP}+\mathrm{FP}}$$

The proposed method SSW-AN shows predictions out of a given sample dataset have more precision than the other existing methods like DCNN, ASDNet, DNN, GCN, and NDN.

**Figure 9:**
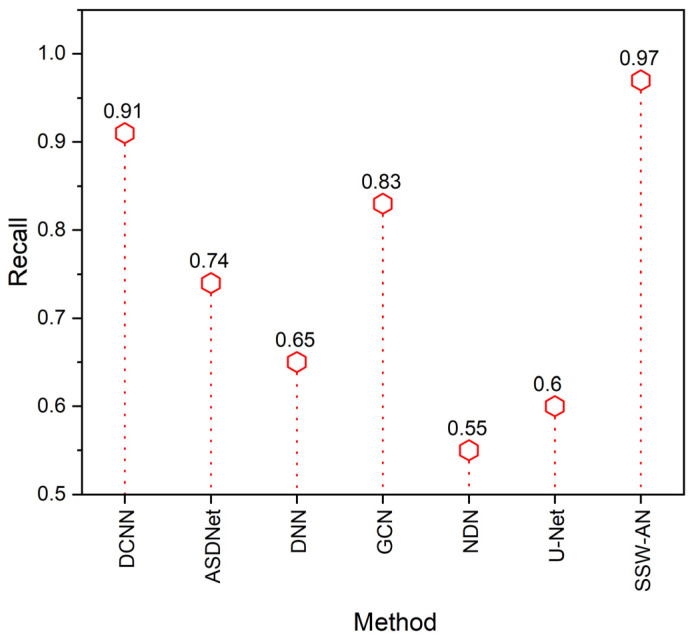
Recall of various methods. Current SSW-AN is compared with DCNN [37], ASDNet [38], DNN [35], GCN [39], NDN [40] and U-Net [41].

Figure 9 shows the comparison of the recall. Recall is a metric used to assess how well information systems that deal with medical imaging can locate the supporting information that a person has needed. The following group of actions has been determined to be essential:

(18) $$\mathrm{Recall}=\frac{\mathrm{TP}}{\mathrm{TP}+\mathrm{FN}}$$

It was found that medical imaging technology, which is the suggested approach of SSW-AN, can track down the supporting data and has a greater recall than the existing methods such as DCNN, ASDNet, DNN, GCN, and NDN.

The term sensitivity refers to the proportion of test samples that are anticipated to return positive results from an experiment. It is a reflection of the scenario with positive samples. The following equation is used to derive the value of sensitivity:

(19) $$\mathrm{Sensitivity}=\frac{\mathrm{TP}}{\mathrm{TP}+\mathrm{FP}}$$

**Figure 10:**
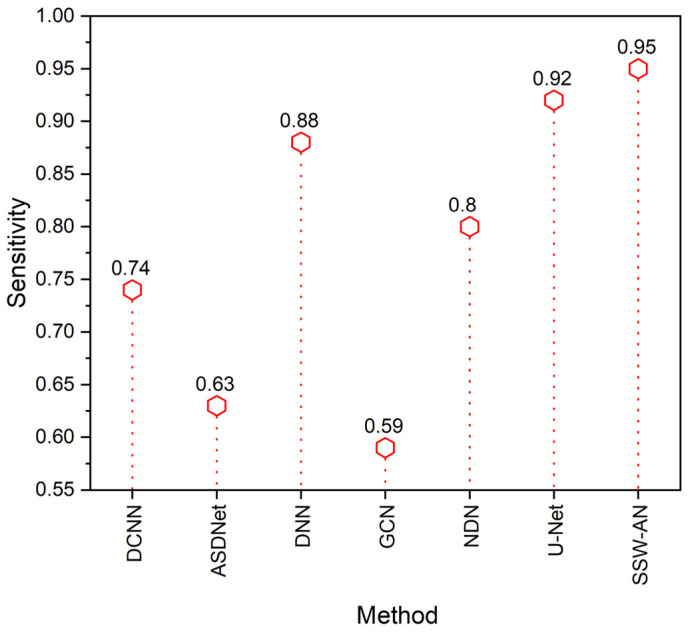
Sensitivity of various methods. Current SSW-AN is compared with DCNN [37], ASDNet [38], DNN [35], GCN [39], NDN [40] and U-Net [41].

Figure 10 depicts the comparison of the sensitivity. The SSW-AN approach provides a higher level of specificity compared to existing methods such as DCNN, ASDNet, DNN, GCN and NDN.

The ability of a classifier to correctly anticipate the real negatives is known as its specificity. This approach stands out due to its precision in identifying Normal instances. Mathematically, this can be expressed as,

(20) $$\mathrm{Specificity}=\frac{\mathrm{TN}}{\mathrm{TP}+\mathrm{FP}}$$

**Figure 11:**
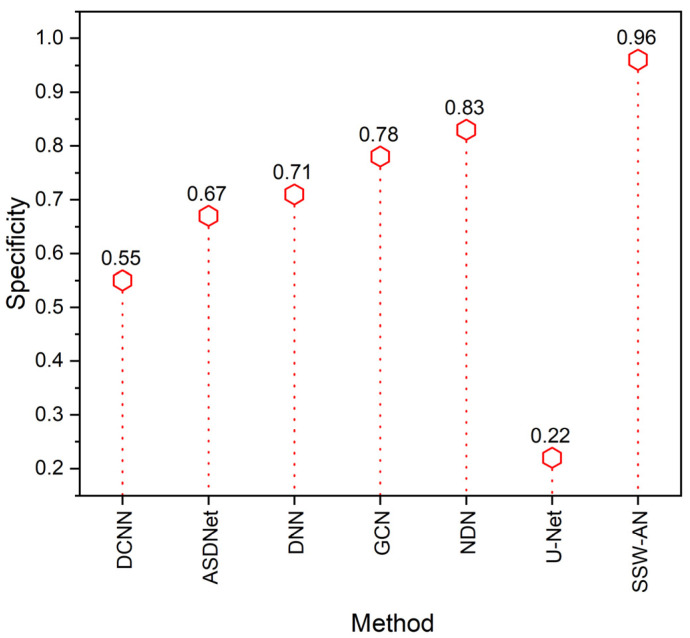
Specificity of various methods. Current SSW-AN is compared with DCNN [37], ASDNet [38], DNN [35], GCN [39], NDN [40] and U-Net [41].

The contrast of the specificity can be seen in Figure 11. The proposed SSW-AN is more specific than other networks, such as DCNN, ASDNet, DNN, GCN and NDN, which are currently being used.

**Figure 12:**
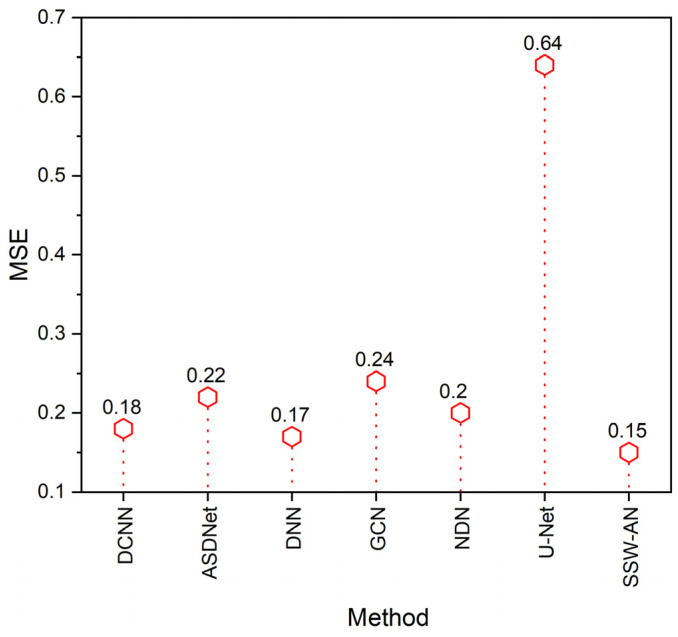
MSE of various methods. Current SSW-AN is compared with DCNN [37], ASDNet [38], DNN [35], GCN [39], NDN [40] and U-Net [41].

The mean squared error (MSE) is a statistical measure used to assess the fit of a regression line to a set of data points. Its worth is proportional to the difference between the current and the previously expected value of the squared error loss. Specifically, it is a risk factor.

(21) $$MSE=\frac{\sum {\left(actual-forecast\right)}^{2}}{k}$$

where:

*k* = total quantity,

Σ = compilation symbol,

The comparison of MSE may be seen in Figure 12. The suggested SSW-AN has the lowest MSE when measured against other methods such as DCNN, ASDNet, DNN, GCN, and NDN. 

### 5.2. Discussion

Parameters demonstrate the suggested method’s superiority over the status quo, which suffers from a number of serious flaws. These are just some of the problems with the current approach. The challenge of the dataset for automatic polyp detection gives us the chance to research cross-data generalizability, which is crucial in the medical field [37]. DNN can create better segmentation masks even for difficult images. To tackle the problem of having too little data to train complicated networks, ASDNet uses fully convolutional adversarial learning and adopts a semi-supervised loss based on attention to certain regions of the image [38]. Although medical image segmentation is still a difficult problem to solve, deep learning applications have shown significant performance gains that may have a positive impact on clinical practice outcomes such as diagnosis [35]. Segmenting images is often critical to several image processing uses. We consider it in terms of a dense categorization issue [39]. One further issue with dice loss is that it is possible to have a false positive because the penalty factor for samples tested is higher than for negative ones [40]. Due to the proposed method’s ability to get over existing method restrictions, we assessed that it is more effective than the existing techniques. U-Net, a technique for segmenting images, was developed especially for this purpose. U-Net has been extensively embraced as the primary tool for segmentation jobs in medical imaging because of its properties, which make it particularly valuable within the community of medical imaging [41]. Medical images also demonstrate that a significant quantity of training data is needed for the DCNN to be effective and that they do not encode the position and orientation of objects. There is no encoding for object orientation or position. They find it difficult to classify images with different perspectives. For network-level data, attention-based semi-supervised learning is not appropriate. The algorithm produces classifications that are less precise than those produced by conventional supervised techniques. Deep neural networks analyze information in complex ways by using sophisticated mathematical modelling, including the need for many learning algorithms and the DNN’s inability to encode object position and orientation. Global Context Network has several applications of image processing, often depending on segmenting pictures. Think about it in terms of a complex classification problem. Nested Dilation Network dice loss has additional drawbacks, including the potential for false positive results due to the greater penalty factor for positive samples than for negative ones. The model’s structure is rigid while training on datasets of various sizes, and the skip connection has not properly used the encoder structure’s properties.

## 6. Conclusions

This research proposed an SSW-AN for semantic picture segmentation. The 2-D wavelet transform, which separates information into low- and high-frequency channels, generates four input and output parameters for the network. This simplifies the teaching process. We use the self-supervised attention module spatial and channel attention subsystems. As a result, the method may be better able to zero in on crucial underlying patterns throughout the channel and the space. Accuracy, precision, recall, sensitivity, and specificity MSE were some of the metrics tested in this experiment. An SSW-AN was proposed, and its results were 98% for accuracy, 97% for precision, 97% for recall, 95% for sensitivity, 96% specificity, and 15% for MSE. The proposed method outperforms the state-of-the-art alternatives. Assessing people with increasingly severe cognitive impairment using the human sickness dataset would remain challenging, given the image’s sensitivity to body movements. Future research should put more emphasis on creating architectures that are still capable of having fewer parameters. As we investigate the feasibility of using a single model for a wide range of applications in medical picture segmentation, we intend to soon simplify the structure without compromising its capacity to create high-quality segmentation masks.

## Figures and Tables

**Table 1:** Performances of the metrics.

Method	Accuracy	Precision	Recall	Sensitivity	Specificity	MSE
DCNN [37]	67%	74%	91%	74%	55%	18%
ASDNet [38]	73%	85%	74%	63%	67%	22%
DNN [35]	83%	64%	65%	88%	71%	17%
GCN [39]	77%	57%	83%	59%	78%	24%
NDN [40]	58%	79%	55%	80%	83%	20%
U-Net [41]	93%	27%	60%	92%	22%	64%
SSW-AN [Proposed]	98%	97%	97%	95%	96%	15%

## Data Availability

Not applicable.
